# High-frequency ultrasound, computed tomography and computed tomography arthrography of the cranial cruciate ligament, menisci and cranial meniscotibial ligaments in 10 radiographically normal canine cadaver stifles

**DOI:** 10.1186/s12917-019-1892-y

**Published:** 2019-05-14

**Authors:** Elke Van der Vekens, Evelien de Bakker, Evelien Bogaerts, Bart J. G. Broeckx, Richard Ducatelle, Kaatje Kromhout, Jimmy H. Saunders

**Affiliations:** 1 Department of Medical Imaging of Domestic Animals and Orthopaedics of Small Animals, Faculty of Veterinary Medicine, Ghent University, Salisburylaan 133, 9820 Merelbeke, Belgium; 2 Department of Nutrition, Genetics and Ethology, Faculty of Veterinary Medicine, Ghent University, Heidestraat 19, 9820 Merelbeke, Belgium; 3 Department of Pathology, Bacteriology and Avian Diseases, Faculty of Veterinary Medicine, Ghent University, Salisburylaan 133, 9820 Merelbeke, Belgium

**Keywords:** CT, Cranial cruciate ligament, Dog, Meniscus, Meniscotibial ligament, Ultrasonography

## Abstract

**Background:**

Bilateral non-traumatic cranial cruciate disease is frequently seen in originally unilateral cruciate pathology. Untreated cranial cruciate ligament disease and concurrent meniscal lesions cause progressive osteoarthritis and pain of the stifle joint. Early presurgical diagnosis is important, but remains difficult.

The purpose of this ex vivo study was (1) to describe the ultrasonographic appearance of the canine cranial cruciate ligament (CrCrL), menisci and meniscal ligaments using a high-frequency linear transducer, (2) to determine the length of the CrCrL seen on ultrasonography (US) and (3) to describe and compare the appearance of the CrCrL, menisci and meniscal ligaments on US, computed tomography (CT) and computed tomography arthrography (CTA).

**Results:**

US and CT examinations were performed on 10 radiographically normal cadaveric stifles of adult dogs weighing more than 15 kg, followed by macroscopic and histologic evaluations. The CrCrL had a parallel hyperechoic fibrillar pattern at the insertion on the tibia and a hypoechoic structure more proximally in all stifles. This pattern was visible over 35% (median) of the total length of the ligament, with 50% (median) of the total length CrCrL that could be outlined. All medial menisci and 8 out of 10 of the lateral menisci showed hypoechoic lines within their bodies oriented obliquely to the direction of the ultrasound beam. Fifteen of the 20 cranial meniscotibial ligaments were detected, showing a hyperechoic fibrillar pattern. Normal macro- and microscopic appearance was observed in all menisci, with the radial bundles of collagen fibers at the level of and with similar orientation as the intrameniscal hypoechoic lines on US.

The CrCrL, menisci and meniscal ligaments were of intermediate density on CT, but marked improvement of the border detection was obtained using CTA. Contrast within the CrCrL was observed in 4/10 stifles using CT and confirmed in 3/4 stifles on histology. One of these ligaments had a partial tear (5–10%) on macroscopic evaluation. None of the menisci showed any abnormalities on CTA.

**Conclusions:**

Normal canine menisci are heterogeneous on high-frequency US and a fibrillar pattern may be observed in the cranial meniscotibial ligaments and the distal portion of the CrCrL. Linear areas of contrast may be detected within the cranial cruciate ligament of radiographically normal stifles.

## Background

Lesions of the cranial cruciate ligament (CrCrL) and menisci are two common causes of stifle lameness, especially in adult, large breed dogs [1, 2]. Non-traumatic or degenerative cranial cruciate disease, frequently occurring bilateral, is seen in 17 to 54% of dogs with originally unilateral disease. This and the fact that some of the dogs that developed contralateral CrCrL rupture had initially normal stifle radiographs, makes early diagnosis important for treatment and prognosis [3–6]. Meniscal pathology is seen in 10 to 83% of the cases concurrent with or subsequent to CrCrL disease. Although meniscal pathology can occur both in the lateral and in the medial meniscus, the medial meniscus is far more affected as it is less mobile than the lateral meniscus [1, 7]. Within both menisci, the axial 75% is poorly vascularized and is commonly affected by lesions, which results in rare spontaneous healing [1, 8, 9]. While various types of injuries can occur, longitudinal tears, usually referred to as bucket-handle tears, are the most common type. These tears often involve the caudal aspect of the medial meniscus and may extend into the meniscotibial ligaments. Meniscal lesions contribute to the progression of osteoarthritis and should therefore be treated [1, 2, 8–10].

CrCrL ruptures are often diagnosed based on clinical findings and radiographs [6]. Studies have shown that in comparison to other imaging modalities like computed tomography arthrography (CTA) and magnetic resonance imaging (MRI), radiography (RX) is less sensitive for early detection of these lesions [4, 11–13]. Similarly, presurgical diagnosis of meniscal lesions during clinical examination or using RX is inconsistent [14, 15]. Until now, studies have described the normal appearance of the canine stifle using RX, ultrasonography (US), computed tomography (CT), CTA and MRI separately [16–21] or have combined the use of several of these techniques [22]. The number of publications specifically on canine stifle US and CTA is however limited. Previous studies on US in normal stifles mentioned that this technique is able to visualize the CrCrL and its hypoechoic appearance. Their conclusion was that only the outline of a limited part of this ligament could be evaluated [18, 23, 24]. Canine menisci are described as triangular structures with a homogeneous echotexture on US [18, 22, 24].

With US and CT becoming widely available and the advances both in terms of hard- and software of ultrasound machines, the authors wanted to investigate their capability to visualize the two most clinically important tissues in the stifle joint in the radiographically normal canine cadaver stifles.

The present study had two main objectives: firstly, to describe the ultrasonographic appearance of the CrCrL, the menisci and meniscal ligaments using a high-frequency linear transducer and to determine the proportion of the cranial cruciate ligament that can be visualized on high-frequency US; Secondly, to describe and compare the cranial cruciate ligament, menisci and meniscal ligaments on high-frequency US, CT, CTA in radiographically normal stifle joints and correlate these observations with macroscopy and histology.

## Results

### Animals

A total of 10 stifles of 5 dog cadavers were collected. These belonged to one of the following breeds: Pitbull, Berger Blanc Suisse, Labrador retriever, Airedale terrier, and Dogo Argentino. Their age ranged from 5 to 13 years old (median: 8 years old), and their weight from 26 to 42.2 kg (median: 38 kg). Three dogs were intact males, along with one neutered male and one a neutered female. None of the dogs had signs of joint effusion or degenerative changes on the orthogonal stifle radiographs.

### Cranial cruciate ligament (CrCrL)

**Fig. 1:**
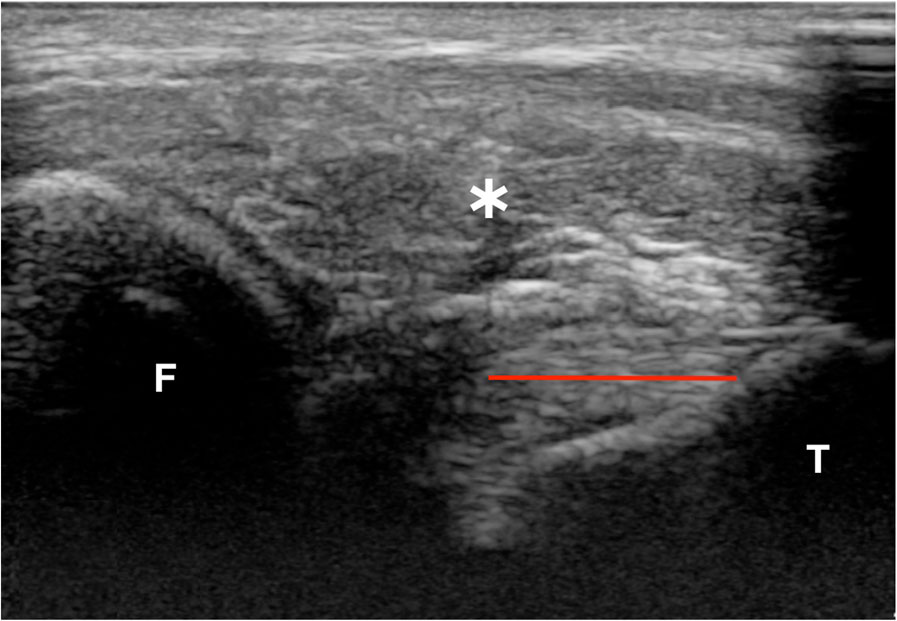
Cranial proximolateral-distomedial oblique sagittal ultrasonographic image of the left stifle of dog 4 in flexion. A clear fibrillar pattern is visible within the distal aspect of the CrCrL. More proximal the ligament becomes hypoechoic because of anisotropy artifact. F: Femur; T: tibia; *: infrapatellar fat pad; red line: length fibrillar pattern visible within CrCrL. Proximal is left and cranial to the top in the image

On US, the CrCrL was seen in all stifles as a hyperechoic fibrillar structure at the insertion on the tibia and more proximally as a hypoechoic structure (Fig. 1). They had smooth and clearly outlined cranial and caudal borders. The parallel fiber pattern was visible over a variable length, with a range of 21 to 48% (median: 35%) of the total length of the ligament (Table 1). The proportion of the CrCrL that could be visualized with US relative to the total length of the ligament (based on CT) ranged from 30 to 60% (median: 50%) (Table 1). No lesions were observed during the US examination.

**Table 1:** The length (in mm) over which parallel fibers in the CrCrL are observed on US (US fibers), the CrCrL can be outlined on US (US outline) and the total length of each CrCrL as measured between both insertions on a dorsal computed tomographic arthrography (CTA) reconstruction

	US fibers	US outline	CTA total length	Weight and breed dog
Dog 1 L	3.95	7.59	15.99	26 kg
Dog 1 R	6.14	9.67	16.82	Pitbull
Dog 2 L	6.65	11.01	20.79	40 kg
Dog 2 R	4.62	6.63	21.89	Berger Blanc Suisse
Dog 3 L	7.80	10.23	18.81	42.2 kg
Dog 3 R	4.83	6.80	18.85	Labrador retriever
Dog 4 L	10.51	10.51	21.70	38 kg
Dog 4 R	9.92	13.17	21.85	Airedale terrier
Dog 5 L	7.04	10.29	19.94	30 kg
Dog 5 R	6.87	8.16	19.67	Dogo Argentino

**Fig. 2:**
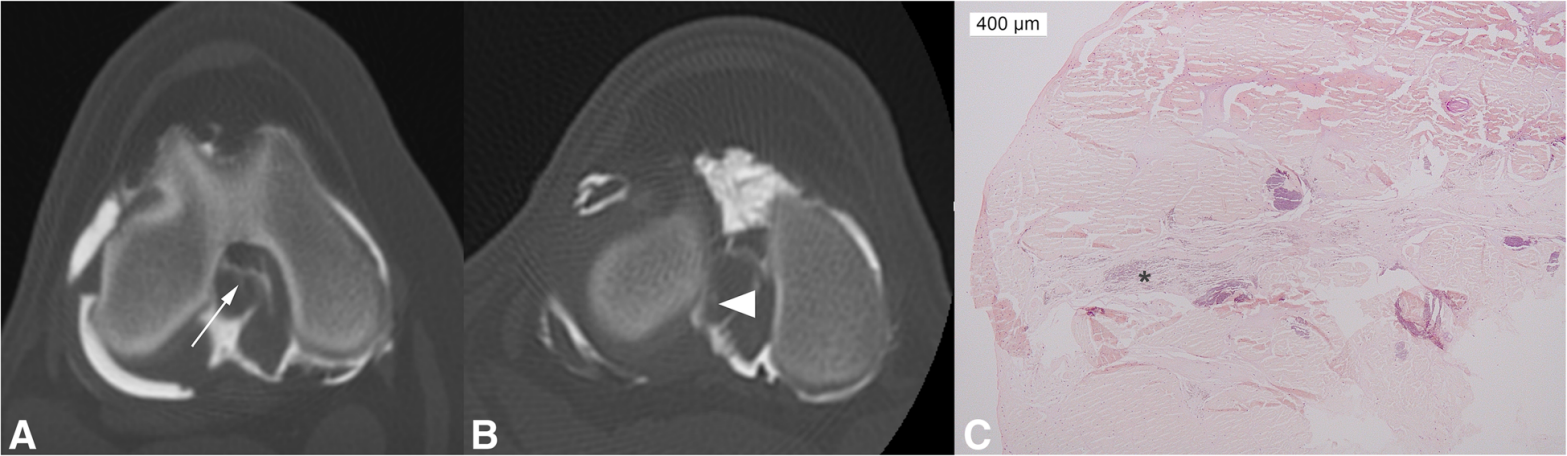
Transverse, CTA image in bone algorithm of the left stifle of dog 2 (**a**) and dog 4 (**b**) using a slice thickness of 0.625 mm. Transverse histological section of the CrCrL of dog 4 (**c**) at the same level after HE staining. Notice the thin contrast line in the mid aspect (arrow) and the caudolateral aspect (arrowhead) of the CrCrL. Note the small purple contrast dots (*) within the less dense collagen tissue between the 2 bundles of the CrCrL and not surrounded by a synovial lining. Lateral is to the left and cranial to the top for images A and B

On CT and CTA, the CrCrL was visible as a poorly (7) to moderately (3) outlined tubular structure with intermediate density (hyperdense to the fat pad) with a marked improved detection of the borders on the post contrast studies in all stifles. No abnormalities were observed on the native images, but in 4 stifles of 3 dogs, a thin linear area of contrast appeared within the ligament which was best seen on the transverse images. In 2 stifles, the contrast was visible in the mid to distal third of the ligament and oriented in a craniocaudal direction, starting at the caudomedial border in both dogs and reaching the mid cranial border of the cranial cruciate ligament in 1 (Fig. 2A). In 2 other stifles, the contrast was detected in the caudolateral aspect of the ligament at the mid third, starting at the caudal border in both cases and even reaching the lateral border in 1 (Fig. 2B).

### Menisci and meniscotibial ligaments

The bodies of both menisci were visible in all modalities (US, CT and CTA).

**Fig. 3:**
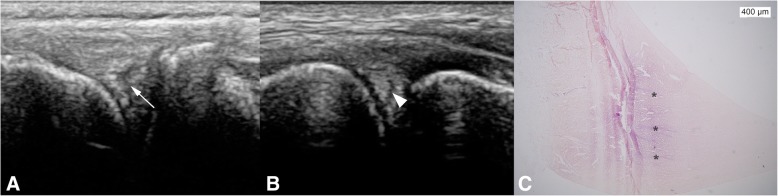
Transverse ultrasonographic image of the lateral meniscus of the left stifle of dog 3 (**a**) and dog 5 (**b**). Transverse histological section of the lateral meniscus of the right stifle of dog 1 (**c**) after HE staining. An S-shaped linear (arrow) or curvilinear (arrowhead) hypoechoic area is visible within the mid aspect of the meniscus and note the purple bundles of collagen (*) representing “tie fibers” running from the abaxial aspect of the meniscus axially

**Fig. 4:**
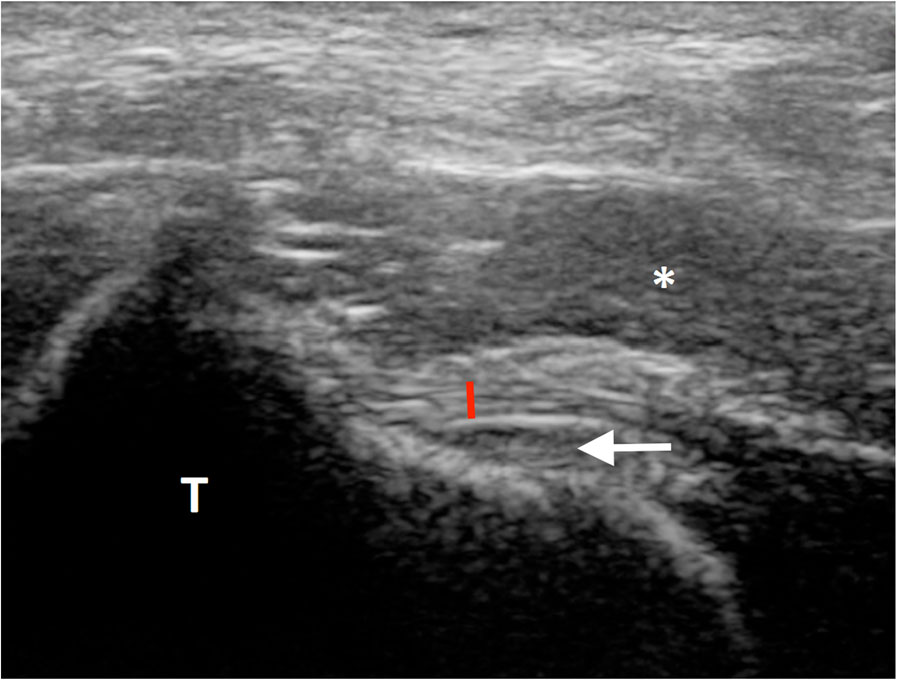
Cranial proximolateral-distomedial oblique sagittal ultrasonographic image of the left stifle of dog 5 in flexion. The tibial insertion of the cranial meniscotibial ligament of the medial meniscus is visible, with a clear fibrillar pattern (red line). The abaxial aspect of the ligament becomes hypoechoic because of the anisotropy artifact. Note the insertion of the CrCrL on the tibia, deep to the cranial meniscotibial ligament of the medial meniscus (arrow). T: tibia; *: infrapatellar fat pad. Lateral is left in the image

On US, the visibility of the medial meniscus was generally better than the lateral one (Table 2). All menisci had a normal shape and position in situ, with the abaxial margin of all menisci aligned with the abaxial margins of the femoral and tibial condyles. All medial menisci and 8 out of 10 lateral menisci had a heterogeneous internal architecture, with hypoechoic lines within the meniscus oriented obliquely to the direction of the ultrasound beam (Fig. 3A, B). The cranial meniscotibial ligament of the medial meniscus was observed in all 10 stifles, whereas the cranial meniscotibial ligament of the lateral meniscus was seen in only 5 out of 10 stifles (Fig. 4). When visualized, the ligaments showed a clear outline of their cranial and caudal borders. Both ligaments had a typical hyperechoic fibrillar structure extending from the cranial horn of the meniscus, with the medial meniscotibial ligament passing cranial to the CrCrL, and the lateral one passing and inserting caudolateral to the CrCrL. The caudal meniscotibial ligaments were not observed on US.

**Table 2:** Ultrasonographic visibility and appearance of the internal structure of the menisci in 10 stifles

	Overall visibility			Aspect internal structure	
Structure	Well	Moderately	Poorly	Homogeneous	Heterogeneous
Lateral meniscus (*n* = 10)	2	3	5	2	8
Medial meniscus (n = 10)	9	1	0	0	10

The bodies and horns of all menisci had a homogeneous appearance on both CT and CTA, with a density similar to the CrCrL. CT allowed for a good visualization of both menisci in all stifles and their outline was graded as moderately visible for all, except for in 1 dog’s images that offered poor visibility of both lateral menisci. Although the cranial meniscotibial ligaments could be localized in all stifles, their outline was unclear in most (poor: 9/10, moderate: 1/10). The caudal meniscotibial ligaments were poorly or not visible at all nor could they be outlined in any of the stifles. On the CTA studies, both the cranial and the caudal meniscotibial ligaments of both menisci were consistently visualized and varied between moderate and well bordered for all stifles. The cranial meniscotibial ligament of the lateral meniscus was generally better outlined than the one of the medial meniscus. The outline of the cranial meniscotibial ligament of the medial meniscus was graded as moderate in 8 and well visible in 2, whereas it was well outlined in 8 of the lateral menisci and moderate in 2. The caudal meniscotibial ligament of the medial meniscus was generally better outlined than that of the lateral meniscus on CTA. It was scored as well visible in 8 stifles and was moderately visible in the 2 others and this for all lateral menisci. CTA improved the outline of 14 menisci (8 lateral and 6 medial), and it remained moderate in 6 (2 lateral and 4 medial). No abnormalities were detected neither in the native, nor in the CTA studies.

### Macroscopy and histology

No abnormalities were detected on macroscopic evaluation of the ligaments, except the CrCrL of the right stifle of dog 5 which showed a partial tear involving 5–10% of its transverse area in its distal third. On histology, contrast particles were observed within the connective tissue between both bundles of the CrCrL in 3 of the 4 stifles where contrast within the CrCrL was detected on CTA (Fig. 2C). The contrast was surrounded by a synovial lining in the right stifle of dog 4. In the 2 other stifles the contrast particles were however free within the less dense connective tissue located between the bundles of otherwise normal cranial cruciate ligaments. These were mainly composed collagen bundles and ligament fibrocytes. There were no reactive cells present surrounding the contrast particles. Mild chondroid metaplasia was observed in the core of both CrCrLs of dog 2. All cranial cruciate ligaments showed a normal synovial lining.

Macroscopically, all menisci were normal. On microscopic evaluation, a normal appearance of the fibrocartilage structure was observed in all menisci, with predominantly circumferentially arranged bundles of collagen fibers. Additional collagen bundles were detected radiating from the abaxial periphery of the meniscus into the inner body, the so-called “tie fibers”. Their presence, extent, direction, and the number of bundles varied between the different menisci. A few blood vessels were observed in the periphery of some of the menisci, adjacent to the radial collagen fibers. These findings were seen in both the lateral as well as the medial menisci.

## Discussion

Previous studies described the CrCrL as a hypoechoic band [18, 21, 22]. They used a 7.5 MHz frequency transducer which is a quite low frequency for scanning small, superficially located structures. Only Seong et al. used a 11 MHz transducer, but they also described the CrCrL as a homogeneous echogenic structure [25]. In our study, we used a 5–17 MHz transducer, mainly using the high-end of the frequency range to create the images. Additionally, an attempt was made in all stifles to orientate the ultrasound beam at an angle of 90° to the long axis of the visualized part of the CrCrL. This allowed for about 50% of the ligament to be outlined in at least half of the stifles on US and, more importantly, a fibrillar pattern was systematically seen in more than a third of the entire length of the CrCrL. These differences in appearance of the CrCrL are most likely due to a combination of multiple factors. Firstly, higher frequencies and technical improvements of the transducers and software allowed for improved and more detailed visualization of small structures [26]. Secondly, anisotropy due to oblique incidence scanning was avoided as much as possible. This has previously been described as an important cause of the hypoechoic appearance of the CrCrL and this artifact was present more proximally where the ligament appeared hypoechoic [16, 25]. Finally, while it was previously difficult to differentiate the hypoechoic infrapatellar fat pad from the CrCrL in non-distended stifles, the observed fibrillar pattern and improved transducers and software made it possible to separate both structures [25, 27]. However, maximal flexion of the stifle was essential to allow perpendicular scanning and visualization of the fibrillar pattern in the CrCrL. Small variations in the degree of flexion, in combination with small irregularities in the skin surface, preventing optimal transducer position, are likely the cause of the variations in the length of the fibrillar pattern observed between CrCrLs of the same dog.

As CrCrL tears are described to originate within the central core of the ligament, the improved visualization of the CrCrL may allow for increased detection of (partial) tears of the CrCrL in the future [2, 10, 28]. Theoretically, it would enable the diagnosis of lesions before they reach the surface and therefore facilitate the detection at an earlier stage than arthroscopy or CTA currently permits. However, more proximal located tears and small peripheral lesions might still be missed as in one CrCrL in this study: a small tear was not detected on US, although it was present in a section that was visible. A possible explanation is that on radiography none of the stifles used in this study showed abnormalities, including the absence of joint effusion. Current literature suggests synovitis to precede fraying of the CrCrL, therefore partial rupture was an unexpected finding and specific search for these lesions was not included in the present protocol [5]. The lack of joint effusion might have caused the loose fibers to stick against the rest of the normal ligament during US thus mimicking normal articular synovium. The presence of fluid around the CrCrL and, even more, a flow in this fluid, causing the ruptured fibers to move, was previously reported as an important factor to increase detection of partial tears on US [25]. Moreover, only a limited part of the ligament (5–10% of the diameter) was torn in this case, possibly being less than the detection limit of the transducer in a non-distended joint. Additionally, these CrCrL were only evaluated in long axis, as this is the described approach to evaluate this structure in dogs, horses and humans [16, 18, 29–31]. However, transverse planes are described to be more sensitive to diagnose small peripheral lesions in equine tendons. Although it will be difficult to obtain images perpendicular to the long axis of the ligament, an attempt could be made to evaluate the distal portion of the CrCrL in a transverse plane. Currently, this approach has only been reported for the proximal aspect of the canine cruciate ligaments by Kramer et al. [18]. As we were able to visualize a fibrillary pattern in the long axis, it is likely that this part of the CrCrL would have a hyperechoic appearance on transverse plane, allowing more detailed evaluation of its entire cross-sectional area. Further research on high-frequency US in stifles with spontaneous partial and complete CrCrL tears and their contralateral stifles in a large number of dogs is necessary to quantify and improve lesion detection. Although in this study, it was one of the stifles showing contrast within the CrCrL on the CTA, no indentation in the outline of the ligament was visible. Incomplete contrast medium outlining the CrCrL or the small defect size obscured by image slice thickness are possible causes for failure of detection of this partial tear on CTA. Small variations in transverse area of normal CrCrL have been previously reported [32]. Also in the horse, mild CrCrL lesions observed on arthroscopy, were not detected on CTA [33].

The visibility and density of the menisci and cruciate ligaments on CT in this study were similar to previous reports, with CTA additionally visualizing the cranial and caudal meniscotibial ligaments and allowing improved definition of the margins of the intra-articular structures [15, 19, 22]. We were able to provide a more detailed description, grading the quality of the outline of these structures and making a comparison between the medial and lateral meniscus and their cranial ligament. The used protocol allowed high quality multiplanar reconstructions in all planes.

While the transverse area of the CrCrL has been described on CTA in dogs by Han et al., the length on CTA, nor the on US visible part of the CrCrL were previously reported [32]. The CrCrL length measured in this study was at the upper limit and slightly above the mean length measured macroscopically in previous studies. This is most likely explained by the relatively larger bodyweight of the large breed dogs used here, compared to previous studies [34, 35].

CTA allowed for a clear outline of the CrCrL in all stifles. The CrCrL is known to be composed out of 2 functional components [2, 10, 35]: a craniomedial portion and a larger caudolateral portion. Although the CrCrL is an intra-articular structure, it is located extrasynovially, being circumferentially covered by a thin synovial sheath [2, 35, 36]. This normally prevents contrast to penetrate into the ligament and/or between both components, as previously reported in human and equine CTA [37, 38]. However, in 4 of the 10 stifles, contrast was detected within the CrCrL on CTA and the direction of the contrast appeared to highlight these 2 components. In addition, on histology, contrast particles were seen within 3 of these 4 ligaments. In one of these 3 stifles, the contrast was surrounded by a synovial lining, suggesting the presence of a synovial fold between both components of the CrCrL. However, in the 2 other stifles the contrast particles were free within this less dense connective tissue. Infiltration of contrast into the CrCrL has been reported in iatrogenic partial CrCrL rupture, but the location of the contrast in this study, both on CTA and histology, makes this an unlikely cause [32]. Many small holes have been observed in the synovial membrane covering the ligament using a scanning electron microscope, but these are too small to allow passage of contrast into the CrCrL [36]. Alternatively, there could have been small tears in the synovium covering the cranial cruciate ligament, allowing contrast to penetrate into the ligament. The synovium is important for the vascularization of the cruciate ligaments and synovial stripping leads to ligament insufficiency [39]. However, a minimal tear was only observed in one of the CrCrL containing contrast in this study. Currently, there are two hypotheses regarding the mechanisms for the occurrence of cruciate ligament fiber damage and synovitis [40, 41]. The first describes synovitis as the primary event, inducing progressive fiber rupture of the cruciate ligaments. However, normal stifle radiographs were a selection criterium in this study, so none showed joint effusion, which is a radiographic sign of synovitis and no such signs were observed on histology. The other hypothesis is that specific factors may induce minor fiber rupture, with subsequent induction of synovitis. Tearing of the synovium surrounding the cranial cruciate ligament may be a primary event responsible for induction of degenerative changes in the CrCrL. A recent study showed histological degeneration of cruciate ligaments without synovitis in more than 25% of dogs with intact cranial cruciate ligaments, but the integrity of the synovial lining was not evaluated [41]. The present study is more compatible with the latter hypothesis as synovial tears were not observed histologically.

To the authors’ knowledge, intraligamentous contrast has not been described in intact CrCrL of animals or humans. Further histological research on a large number of normal and pathological CrCrLs is therefore necessary to determine the exact origin of the intraligamentous contrast and to differentiate an early sign of pathology from a, previously undescribed, anatomical variant.

Little et al. have reported significant synovitis and fraying of the CrCrL in stifles with minimal radiographic joint effusion and a partial tear was seen in one of the CrCrL of this study [13]. These findings, as also stated in previous studies, confirm that normal stifle radiographs do not exclude partial CrCrL ruptures [4, 11, 12]. This knowledge should be taken into account in both clinical and research settings as the absence of radiographic findings is often mistaken for (presumed) normality.

Mild chondroid metaplasia was observed in both CrCrLs of one dog and intraligamentous contrast was observed in one of its stifles, both on CTA and histology. Vasseur et al. reported that, in dogs of 5+ years of age and with a bodyweight of 15+ kg, ligaments with chondroid metaplasia on hematoxylin-eosin-stained sections have reduced mechanical properties [42]. More recently, mild chondroid metaplasia has been described in the CrCrLs of normal Labrador Retrievers and Greyhounds. The authors proposed this degeneration was a physiological adaptation to repetitive stress [43]. The changes seen in the dog in our study can be compatible with both age-related physiological changes or an early form of pathological degeneration.

The purpose of this study was to describe the differences in appearance of the structures compared to the in literature generally accepted description and not to compare cases and controls. Additional studies using and comparing high-frequency US and CTA of the stifle of dogs with and without CrCrL disease, are necessary to determine the accuracy of lesion detection with these techniques.

This study is the first description of the ultrasonographic appearance of cranial meniscotibial ligaments, as these structures were not identified in previous US studies of canine stifles [18, 22]. This was also most likely due to the lower frequency used during previous examinations, which limits the visualization of small superficial structures [7, 26]. Arnault et al. described high-frequency US as a reliable means of assessing menisci, superficial tendons and superficial ligaments in the stifle, but reported meniscal ligaments to be invisible [27]. In this study, we were able to visualize the cranial meniscotibial ligaments in every stifle medially and in half of the stifles laterally and all showed a fibrillary pattern. Therefore, we have provided a detailed protocol that can be used to visualize those ligaments in future studies. Although lesions most commonly involve the caudal meniscotibial ligament of the medial meniscus, meniscal tears into the cranial horn and cranial meniscotibial ligament have also been reported [9, 44]. Improved visualization of the cranial horn of the meniscus and the meniscotibial ligament could facilitate presurgical diagnosis of meniscal lesions.

The lateral meniscus was more difficult to visualize compared to the medial one. This is in agreement with previous reports that described that the mid and caudal part of the meniscus were partially obscured by the fibular head [21, 24].

The menisci have generally been reported as homogeneous triangular structures with moderate echogenicity [7, 18, 22], with the exception of one study of Marino et al. where they were reported to be heterogeneous, but no images or extra details were provided in that study [45].

In our study, however, nearly all menisci showed a heterogeneous appearance, with both hypoechoic lines and larger hypoechoic areas visible within their body. Several authors have described this as one of the signs of meniscal tears [7, 18, 27, 46], but in spite of that, none of the menisci in our study showed any macroscopic or histological abnormality using the OARSI grading systems [8, 47]. Because these hypoechoic areas and lines are starting both within the structure and at the periphery of the meniscus and they are running in an oblique direction to the ultrasound beam, it is unlikely they represent artefacts. Kambic et al. have described the presence of radial “tie fibers” on microscopic specimen of the canine menisci [48]. As these bundles of collagen fibers were also observed histologically in the menisci of this study and their location and direction was compatible with the observed changes within the menisci on US, they are a logical explanation for the hypoechoic lines. As these lines are thin, the increased resolution associated with using high-frequency US in this study likely explains their visibility. Radial tie fibers have also been reported in high field MRI by Li et al. as a cause for linear high signal intensity areas in the central layer of human menisci [49]. These tie fibers were considered a possible cause of false positive diagnosis of meniscal tears [49, 50]. This matches with findings at our animal hospital, where menisci with normal shape, but heterogeneous internal structure have been frequently observed during stifle US in patients with stifle pathology, but without meniscal lesions on arthroscopy or mini-arthrotomy.

All menisci were normal and homogeneous on CT or CTA. Although the slice thickness of 0.625 mm used in this study was less than the previously reported 1 mm slices in canine stifles [22, 51], it was approximately 6 to 10 times thicker than the thickness of the hypoechoic lines observed on US in this study. Considering that the entire meniscus is mainly composed of collagen, it is unlikely that CT would be able to detect these density differences in a clinical setting [48]. No contrast was detected within the menisci thus indicating integrity of their surfaces, which was confirmed macro- and microscopically and is similar to previous reports [8].

Better lesion detection in menisci has been reported with dorsal plane CT arthrography than in the transverse plane [15, 38]. Regardless of this fact, multiplanar reconstructions will be necessary to optimize the visualization of other structures such as cruciate ligaments [32]. In the present study, thinner transverse slices were used allowing for high quality dorsal reconstructions, thereby combining good detail in both cruciate ligaments and menisci.

The stifles in this study were positioned in caudal recumbency. This position was preferred because in patients, dorsal recumbency more easily allows for extended stifle position with tibial plateau oriented parallel to the gantry.

The volume and concentration of contrast used for CTA are similar to the ones used in previous studies and clearly outlined the cranial cruciate ligament in all dogs [19, 32, 51]. However, the menisci and cranial meniscotibial ligaments were not completely surrounded by contrast in all stifles, explaining the variation in their outline. This might be improved by increasing the volume to 3–6 mL, as advised in clinical patients [51].

## Conclusions

In dogs weighing more than 15 kg, high-frequency US allows visualization of a fibrillar pattern in the distal third of the cranial cruciate ligament and the cranial meniscotibial ligaments. Normal canine menisci are heterogeneous on high-frequency US.

CTA and high-frequency US are compatible for detailed evaluation of the canine CrCrLs, menisci and cranial meniscotibial ligaments with both techniques possibly enabling earlier presurgical lesion detection.

Linear areas of contrast may be detected within the cranial cruciate ligament of radiographically normal stifles, but further research is necessary to determine the origin and clinical significance of these findings.

## Methods

### Animals

Included dogs died or were euthanized at the small animal clinic of Ghent University for reasons unrelated to diseases of the stifle joint. Necropsy and further research was performed with the owners’ consent. This study involves a work on dog cadavers, using euthanized animals that had not been included in a procedure before, thus not requiring prior ethical approval based on Belgian and European legislation (EU directive 2010/63/EU).

Inclusion criteria were a minimum weight of 15 kg, the availability of information on the age, sex and breed and the absence of radiographic abnormalities (see further). The sample size was determined based on similar published research [52, 53]. The hind limbs were disarticulated in the coxofemoral joint within the first 24 h post mortem, with the cadavers stored at 7 °C and all procedures performed within 60 h post mortem. The stifles were evaluated radiographically on survey orthogonal radiographs to confirm normal appearance. A DR system with Cesium Iodide (CsI) detector (DX-D 40C; AGFA) was used with exposure values of 52 to 54 kVp and 16–25 mAs.

### Ultrasonographic examination

All stifles were prepared for US examination by clipping the hair from mid femur to mid tibia and applying isopropyl alcohol and ultrasound coupling gel. The US examination was performed by a board-certified radiologist (EVDV) using a linear phased array probe with a frequency range of 5–17 MHz (Phillips iU22). The CrCrL and the menisci were visualized as described previously [18].

The cranial meniscotibial ligaments were observed using a protocol similar to the one described in horses [29]. The stifle was maximally flexed. The cranial third of respectively the lateral or medial meniscus was scanned with the probe oriented in a proximodistal direction, visualizing the meniscus as a triangular structure. The meniscus was followed cranially, the probe remaining perpendicular to its abaxial surface until it became more ovoid in shape. At this level, the probe was rotated in a proximoabaxial-distoaxial direction over ±80°, visualizing the cranial meniscotibial ligaments as thin structures with a longitudinal parallel fibrillar pattern. Keeping this oblique orientation and sliding the probe slightly craniodistally, the ligaments could be followed to their insertion on the cranial intercondylar area of the tibia. The meniscotibial ligament of the medial meniscus was visualized cranial to the distal attachment site of the CrCrL and the ligament of the lateral meniscus was inserting caudal to it.

Images allowing maximal visualization of the CrCrL were stored and reviewed using available Osirix software (Pixmeo Sarl). The evaluation criteria for the menisci, cranial meniscotibial ligaments and CrCrL during US examination are described in Table 3.

**Table 3:** Evaluation criteria used for ultrasound, computed tomography (CT) and computed tomography arthrography (CTA) to evaluate the menisci, meniscotibial ligaments and cranial cruciate ligament (CrCrL)

Structure	Criteria	Ultrasound	CT	CT-arthrography
Meniscus	Overall visibility	0: not visible 1: poorly visible 2: moderately visible 3: well visible	0: not visible 1: poorly visible 2: moderately visible 3: well visible	0: not visible 1: poorly visible 2: moderately visible 3: well visible
	Aspect internal structure	0: heterogeneous 1: homogeneous (for visualised part)	0: heterogeneous 1: homogeneous	0: heterogeneous 1: homogeneous
	Outline	0: not triangular and smooth 1: triangular and smooth	0: not visible 1: poorly visible 2: moderately visible 3: well visible	0: not visible 1: poorly visible 2: moderately visible 3: well visible
Cranial menisco-tibial ligaments	Overall visibility	0: not visible 1: poorly visible 2: moderately visible 3: well visible	0: not visible 1: poorly visible 2: moderately visible 3: well visible	0: not visible 1: poorly visible 2: moderately visible 3: well visible
	Aspect internal structure	0: no longitudinal echoes detected 1: moderate longitudinal echo pattern 2: clear longitudinal echo pattern over entire width ligament	0: heterogeneous 1: homogeneous	0: heterogeneous 1: homogeneous
	Outline	0: not visible 1: poorly visible 2: moderately visible 3: well visible	0: not visible 1: poorly visible 2: moderately visible 3: well visible	0: not visible 1: poorly visible 2: moderately visible 3: well visible
Caudal menisco-tibial ligaments	Overall visibility	0: not visible 1: visible	0: not visible 1: poorly visible 2: moderately visible 3: well visible	0: not visible 1: poorly visible 2: moderately visible 3: well visible
	Aspect internal structure	/	0: heterogeneous 1: homogeneous	0: heterogeneous 1: homogeneous
	Outline	/	0: not visible 1: poorly visible 2: moderately visible 3: well visible	0: not visible 1: poorly visible 2: moderately visible 3: well visible
Cranial cruciate ligament	Visibility / structure identification	0: not visible 1: visible	0: not visible 1: poorly visible 2: moderately visible 3: well visible	0: not visible 1: poorly visible 2: moderately visible 3: well visible
	Visibility Outline	0: not visible 1: poorly visible 2: moderately visible 3: well visible	0: not visible 1: poorly visible 2: moderately visible 3: well visible	0: not visible 1: poorly visible 2: moderately visible 3: well visible
	Length outline visualized	In mm and % of total length CrCrL on CTA	/	Total length CrCrL in mm
	Smoothness of outline	0: irregular 1: smooth	/	0: irregular 1: smooth
	Aspect internal structure	0: no longitudinal echoes detected 1: moderate fibrillar pattern 2: clear fibrillar pattern over entire width ligament	0: heterogeneous 1: homogeneous	0: heterogeneous with contrast within ligament 1: homogeneous
	Length longitudinal echoes visualized	In mm and % of total length CrCrL on CTA	/	/

The length of the CrCrL was defined as the distance in millimeters over which the cranial and caudal borders of the ligament were visualized on US from the insertion on the tibia and included both the hyper- and hypoechoic parts of the CrCrL. The proportion of the CrCrL that could be visualized was measured relative to the total length as observed on CT.

### Computed tomographic (CT) and CT arthrographic examination

For the CT examination, all data was collected using a four-slice CT scanner (Lightspeed QX, GE Medical systems, USA). The stifles were positioned with the caudal aspect of the limb on a CT cushion, simulating a caudal extended position of the hind limbs in dogs in dorsal recumbency. The tibial plateau was positioned parallel to the scanning plane and this was confirmed on the scout view. Overlapping transverse pre-arthrography CT images of 1.25 mm were obtained from the distal third of the femur to the proximal third of the tibia/fibula, using a bone algorithm, 100 kVp, 140 mA, a field of view of 96 mm and a pitch of 0.75. A second scan was performed from the proximal aspect of the patella to the tibial plateau, with a slice thickness of 0.625 mm thickness. These images were acquired in a bone algorithm, 100 kVp, 180 mA, a field of view of 96 mm and a pitch of 1.

Arthrocentesis was performed with a 23-gauge needle inserted lateral to the mid-point of the patellar ligament. Iohexol (Omnipaque 240) was injected using a volume of 2 to 2.4 mL (median 2.2 mL) at a concentration 120 mg I/mL. The joint was flexed and extended repeatedly to ensure even distribution of contrast. The limb was repositioned on the CT cushion as before the contrast injection. The CT protocol was repeated for all stifles after intra-articular positive contrast injection.

All CT and CTA images were reviewed by the same radiologist (EVDV), using the available image viewer.

To evaluate the CrCrL, mainly transverse slices parallel to the tibial plateau were used. However, sagittal and dorsal reconstructions along and transverse reconstructions perpendicular to the long axis of the ligament were created in all stifles. The length of each cranial cruciate ligament was measured on the dorsal reconstruction from the center of the tibial attachment to the center of the femoral attachment using the available measurement tool.

The menisci and meniscotibial ligaments were evaluated using the transverse slices and dorsal and sagittal reconstructions. All multiplanar reconstructions were made using the 0.625 mm slice thickness CTA with available Osirix software (WL: 500; WW: 3500).

The CrCrL and the menisci were evaluated for the presence of abnormalities or findings not previously reported in normal CrCrL and menisci.

An overview of all evaluation criteria used during US, CT and CTA is given in Table 3, focusing on their visibility, outline and internal structure.

### Macroscopic and microscopic dissection

For macroscopic dissection the hindlimbs were positioned as if dogs were in dorsal recumbency, with the stifle joint in a 90° flexed position. A n°15 blade bistoury was used to make a curved parapatellar skin incision extending from the patella to the tibial tuberosity. The subcutaneous fascia was incised and retracted with the skin. The patellar ligament was cut just proximal to its attachment to the tibial tuberosity and retracted in order to inspect the patella and patellar groove. The proximal attachment of the extensor digitorum longus tendon and the lateral and medial collateral ligaments were carefully dissected to provide a more detailed approach of the stifle joint. The CrCrL was cut proximally and its distal attachment was carefully dissected from the intermeniscal and cranial tibial ligament of the medial meniscus. The medial and lateral menisci were dissected close to the bone with preservation of the cranial and caudal tibial ligament of both menisci and the femoral ligament of the lateral meniscus. The intermeniscal ligament was also dissected during this action. The caudal cruciate ligament was dissected at its proximal and distal attachment. The cranial and caudal cruciate ligaments and the medial and lateral menisci were collected and fixed in 4% paraformaldehyde solution for further histopathologic examination. The remnants of the stifle joints were wrapped and preserved in a − 18 °C freezer.

Histologically, after the meniscus was fixated with 10% neutral buffered formalin, coronal sections were prepared, cut at 90° to the longitudinally oriented collagen fibers and taken from the mid body of the meniscus. Following fixation, the CrCrLs were first separated in a proximal, mid and distal segment. Each of these segments was also cut into serial sections perpendicular to the longitudinally oriented collagen fibers. Cell count and morphology of the meniscus and CrCrL were investigated after hematoxylin and eosin staining and the tissues were observed under a light microscope.

All US, CT and CTA images were independently evaluated, blinded to the macroscopic and histology findings.

## Abbreviations

CrCrL: cranial cruciate ligament
CT: computed tomography
CTA: computed tomography arthrography
MRI: magnetic resonance imaging
RX: radiography
US: ultrasonography

## References

[1] Flo GL (1993). Meniscal injuries. Vet Clin N Am-Small Anim Pract.

[2] Vasseur PB, Slatter D (2003). Stifle Joint. Textbook of small animal surgery.

[3] Doverspike M, Vasseur PB, Harb MF, Walls CM (1993). Contralateral cranial cruciate ligament rupture: incidence in 114 dogs. J Am Anim Hosp Assoc.

[4] de Bruin T, de Rooster H, Bosmans T, Duchateau L, van Bree H, Gielen I (2007). Radiographic assessment of the progression of osteoarthritis in the contralateral stifle joint of dogs with a ruptured cranial cruciate ligament. Vet Rec..

[5] Muir P, Schwartz Z, Malek S, Kreines A, Cabrera SY, Buote NJ (2011). Contralateral cruciate survival in dogs with unilateral non-contact cranial cruciate ligament rupture. PLoS One.

[6] Chuang C, Ramaker MA, Kaur S, Csomos RA, Kroner KT (2014). Radiographic risk factors for contralateral rupture in dogs with unilateral cranial cruciate ligament rupture. PLoS One.

[7] Mahn MM, Cook JL, Reeves Cook C, Balke MT (2005). Arthroscopic verification of ultrasonographic diagnosis of meniscal pathology in dogs. Vet Surg.

[8] Jackson J, Vasseur PB, Griffey S, Walls CM, Kass PH (2001). Pathologic changes in grossly normal menisci in dogs with rupture of the cranial cruciate ligament. J Am Vet Med Assoc.

[9] Ralphs SC, Whitney WO (2002). Arthroscopic evaluation of menisci in dogs with cranial cruciate ligament injuries: 100 cases (1999-2000). J Am Vet Med Assoc.

[10] Fossum TW, Hedlund CZ, Johnson AL, Schulz KS, Seim HBIII, Willard MD, Fossum TW, Dewey CW, Horn CV, Johnson AL, CM MP, MAG R (2007). Diseases of the joints. Small animal surgery.

[11] D’Anjou M-A, Moreau M, Troncy E, Martel-Pelletier J, Abram F, Raynauld J-P, Pelletier J-P (2008). Osteophytosis, subchondral bone sclerosis, joint effusion and soft tissue thickening in canine experimentail stifle osteoarthritis: comparison between 1.5T magnetic resonance imaging and computed radiography. Vet Surg.

[12] Bogaerts E, Van der Vekens E, Verhoeven G, de Rooster H, Van Ryssen B, Samoy Y (2018). Intraobserver and interobserver agreement on the radiographical diagnosis of canine cranial cruciate ligament rupture. Vet Rec.

[13] Little JP, Bleedorn JA, Sutherland BJ, Sullivan R, Kalscheur VL, Ramaker MA (2014). Arthroscopic assessment of stifle synovitis in dogs with cranial cruciate ligament rupture. PLoS One.

[14] Case JB, Hulse D, Kerwin SC, Peycke LE (2008). Meniscal injury following initial cranial cruciate ligament stabilization surgery in 26 dogs (29 stifles). Vet Comp Orthop Traumatol.

[15] Tivers MS, Mahoney PN, Baines EA, Corr SA (2009). Diagnostic accuracy of positive contrast computed tomography arthrography for the detection of injuries to the medial meniscus in dogs with naturally occurring cranial cruciate ligament insufficiency. J Small Anim Pract.

[16] Reed AL, Payne JT, Constantinescu GM (1995). Ultrasonographic anatomy of the normal canine stifle. Vet Radiol Ultrasound..

[17] Baird DK, Hathcock JT, Rumph PF, Kincaid SA, Visco DM (1998). Low-field magnetic resonance imaging of the canine stifle joint: normal anatomy. Vet Radiol Ultrasound..

[18] Kramer M, Stengel H, Gerwing M, Schimke E, Sheppard C (1999). Sonography of the canine stifle. Vet Radiol Ultrasound..

[19] Samii VF, Dyce J (2004). Computed tomographic arthrography of the normal canine stifle. Vet Radiol Ultrasound..

[20] Konar M, Kneissl S, Vidoni B, Lang J, Mayrhofer E (2005). Niederfeld-Magnetresonanztomographie am Kniegelenk des Hundes. Teil 1: Untersuchungsprotokolle und Sequenzen. Tierarztl Prax Ausg K Kleintiere Heimtiere..

[21] Cook CR (2016). Ultrasound imaging of the musculoskeletal system. Vet Clin N Am-Small Anim Pract..

[22] Soler M, Murciano J, Latorre R, Belda E, Rodriguez MJ, Agut A (2007). Ultrasonographic, computer tomographic and magnetic resonance imaging anatomy of the normal canine stifle joint. Vet J.

[23] Gnudi G, Bertoni G (2001). Echographic examination of the stifle joint affected by cranial cruciate ligament rupture in the dog. Vet Radiol Ultrasound.

[24] Nayseh K, Kramer M, Ondreka N (2015). Ultrasonographic examination of the stifle joint in the dog. Part 1: Ultrasonographic anatomy, standardized scanning protocol and common indications. Tierarztl Prax Ausg K Kleintiere Heimtiere.

[25] Seong Y, Eom K, Lee H, Lee J, Park J, Lee K (2005). Ultrasonographic evaluation of cranial cruciate ligament rupture via dynamic intra-articular saline injection. Vet Radiol Ultrasound..

[26] Gatel L, Guillaume G, Chalvet-Monfray K, Saunders JH, Rault DN (2016). Intra- and inter-observer variability in ultrasonographical measurements of uteri and ovaries in healthy, non-pregnant queens. J Feline Med Surg.

[27] Arnault F, Cauvin E, Viguier E, Kraft E, Sonet J, Carozzo C (2009). Diagnostic value of ultrasonography to assess stifle lesions in dogs after cranial cruciate ligament rupture: 13 cases. Vet Comp Orthop Traumatol.

[28] Whitehair JG, Vasseur PB, Willits NH (1993). Epidemiology of cranial cruciate ligament rupture in dogs. J Am Vet Med Assoc.

[29] Hoegaerts M, Saunders JH (2004). How to perform a standardized ultrasonographic examination of het equine stifle. Proc Am Ass equine Practnrs.

[30] Suzuki S, Kasahara K, Futami T, Iwasaki R, Ueo T, Yamamuro T (1991). Ultrasound diagnosis of pathology of the anterior and posterior cruciate ligaments of the knee joint. Arch Orthop Trauma Surg.

[31] Chen P-T, Wu C-H, Yu C-W, Wang J-H, Shih T-F, Wang T-G (2013). Sonography of the normal anterior cruciate ligament: a preliminary report. J Med Ultrasound.

[32] Han S, Cheon H, Cho H, Kim J, Kang J-H, Yang M-P (2008). Evaluation of partial cranial cruciate ligament rupture with positive contrast computed tomographic arthrography in dogs. J Vet Sci.

[33] Nelson BB, Kawcak CE, Goodrich LR, Werpy NM, Valdes-Martinez A, McIlwraith CW (2016). Comparison between computed tomographic arthrography, radiography, ultrasonography, and arthroscopy for the diagnosis of femorotibial joint disease in western performance horses. Vet Radiol Ultrasound..

[34] de Rooster H, de Bruin T, van Bree H (2006). Morphological and functional features of the canine cruciate ligaments. Vet Surg.

[35] de Rooster H, de Bruin T, van Bree H. Morphology and function of the cruciate ligaments. In: Muir P, editor. Advances in the canine cranial cruciate ligament. Iowa: Wiley-Blackwell; 2010. p. 5–12.

[36] Kobayashi S, Baba H, Uchida K, Negoro K, Sato M, Miyazaki T (2006). Microvascular system of anterior cruciate ligament in dogs. J Orthop Res.

[37] Buckwalter KA (2006). CT arthrography. Clin Sports Med.

[38] Tivers MS, Mahoney P, Corr SA (2008). Canine stifle positive contrast computed tomography arthrography for assessment of caudal horn meniscal injury: a cadaver study. Vet Surg.

[39] Robinson D, Halperin N, Nevo Z (1992). Devascularization of anterior cruciate ligament by synovial stripping in rabbits. Acta Orthop Scand.

[40] Bleedorn JA, Greuel EN, Manley PA, Schaefer SL, Markel MD, Holzman G (2011). Synovitis in dogs with stable stifle joints and incipient cranial cruciate ligament rupture: a cross-sectional study. Vet Surg.

[41] Döring A-K, Junginger J, Hewicker-Trautwein M (2018). Cruciate ligament degeneration and stifle joint synovitis in 56 dogs with intact cranial cruciate ligaments: correlation of histological findings and numbers and phenotypes of inflammatory cells with age, body weight and breed. Vet Immunol Immunopathol.

[42] Vasseur PB, Pool RR, Arnoczky SP, Lau RE (1985). Correlative biomechanical and histological study of the cranial cruciate ligament in dogs. Am J Vet Res.

[43] Comerford EJ, Tarlton JF, Wales A, Bailey AJ, Innes JF (2006). Ultrastructural difference in cranial cruciate ligaments from dogs of two breeds with a differing predisposition to ligament degeneration and rupture. J Comp Pathol.

[44] Martig S, Konar M, Schmökel HG, Rytz U, Spreng D, Scheidegger J (2006). Low-field MRI and arthroscopy of meniscal lesions in ten dogs with experimentally induced cranial cruciate ligament insufficiency. Vet Radiol Ultrasound..

[45] Marino DJ, Loughin CA (2010). Diagnostic imaging of the canine stifle: a review. Vet Surg.

[46] Franklin SP, Cook JL, Cook CR, Shaikh LS, Clarke KM, Holmes SP (2017). Comparison of ultrasonography and magnetic resonance imaging to arthroscopy for diagnosing medial meniscal lesions in dogs with cranial cruciate ligaments deficiency. J Am Vet Med Assoc.

[47] Cook JL, Kuroki K, Visco D, Pelletier J-P, Schulz L, Lafeber FPJG (2010). The OARSI histopathology initiative – recommendations for histological assessments of osteoarthritis in the dog. Osteoarthr Cartil.

[48] Kambic HE, McDevitt CA (2005). Spatial organization of types I and II collages in the canine meniscus. J Orthop Res.

[49] Li CA, Kim MK, Kim IH, Lee JH, Jang KY, Lee SY (2013). Correlation of histological examination of meniscus with MR images: focused on high signal intensity of the meniscus not caused by definite meniscal tear and impact on MR diagnosis of tears. Korean J Radiol.

[50] Hauger O, Frank LR, Boutin RD, Lektrakul N, Chung CB, Haghighi P (2000). Characterisation of the “red zone” or knee meniscus: MR imaging and histologic correlation. Radiology..

[51] Samii VF, Dyce J, Pozzi A, Drost WT, Mattoon JS, Green EM (2009). Computed tomographic arthrography of the stifle for detection of cranial and caudal cruciate ligament and meniscal tears in dogs. Vet Radiol Ultrasound..

[52] Santos MP, Gutierrez-Nibeyro SD, McKnight AL, Singh K (2015). Gross and histopathologic correlation of low-field magnetic resonance imaging findings in the stifle of asymptomatic horses. Vet Radiol Ultrasound..

[53] Daglish J, Frisbie DD, Selberg KT, Barrett MF (2018). High field magnetic resonance imaging is comparable with gross anatomy for description of the normal appearance of soft tissues in the equine stifle. Vet Radiol Ultrasound..

